# *Streptococcus agalactiae* carriage among pregnant women living in Rio de Janeiro, Brazil, over a period of eight years

**DOI:** 10.1371/journal.pone.0196925

**Published:** 2018-05-11

**Authors:** Ana Caroline N. Botelho, Juliana G. Oliveira, Andreia P. Damasco, Késia T. B. Santos, Ana Flávia M. Ferreira, Gabriel T. Rocha, Penélope S. Marinho, Rita B. G. Bornia, Tatiana C. A. Pinto, Marco A. Américo, Sergio E. L. Fracalanzza, Lúcia M. Teixeira

**Affiliations:** 1 Departamento de Microbiologia Médica, Instituto de Microbiologia Paulo de Góes, Universidade Federal do Rio de Janeiro, Rio de Janeiro, Rio de Janeiro, Brazil; 2 Hospital Maternidade Escola, Universidade Federal do Rio de Janeiro, Rio de Janeiro, Rio de Janeiro, Brazil; Universidade de Lisboa Faculdade de Medicina, PORTUGAL

## Abstract

Group B *Streptococcus* (GBS) carriage by pregnant women is the primary risk factor for early-onset GBS neonatal sepsis. Intrapartum antibiotic prophylaxis (IAP) can prevent this transmission route, and two main approaches are recommended to base the selection of pregnant women to be submitted to IAP: the risk-based and the culture-based strategies. In Brazil, compliance to such recommendations is poor, and not much is known about GBS carriage. In the present study, 3,647 pregnant women living in Rio de Janeiro State, Brazil, were screened for GBS anogenital colonization, over a period of 8 years (2008–2015). GBS was detected in 956 (26.2%) of them, and presence of vaginal discharge was the only trait associated with a higher risk for GBS colonization. Serotypes Ia (257; 37.3%) and II (137; 19.9%) were the most frequent among 689 (72.1% of the total) GBS isolates evaluated, followed by NT isolates (84; 12.1%), serotype Ib (77; 11.1%), V (63; 9.1%), III (47; 6.8%) and IV (24; 3.5%). Estimated coverage of major serotype-based GBS vaccines currently under clinical trials would vary from 65.2% to 84.3%. All 689 isolates tested were susceptible to ampicillin and vancomycin. Resistance to chloramphenicol, clindamycin, erythromycin, levofloxacin, and tetracycline was observed in 5% (35), 2% (14), 14% (97), 5% (35) and 86% (592) of the isolates, respectively. No significant fluctuations in colonization rates, serotype distribution and antimicrobial susceptibility profiles were observed throughout the period of time investigated. The culture-based approach for IAP recommendation showed to be the best choice for the population investigated when compared to the risk-based, since the first did not increase the number of pregnant women submitted to antibiotic therapy and covered a larger number of women who were actually colonized by GBS. The fact the not all isolates were available for additional characterization, and serotype IX antiserum was not available for testing represent limitations of this study. Nevertheless, to the best of our knowledge, this is the largest investigation on GBS carriage among pregnant women in Brazil up to date, and results are useful for improving GBS prevention and treatment strategies.

## Introduction

*Streptococcus agalactiae* (Group B *Streptococcus*, GBS) remains as a major cause of morbidity and mortality among newborns in many countries [1]. Pregnant women asymptomatically colonized by GBS in the genitourinary and/or gastrointestinal tracts are the main reservoir of this microorganism, and early-onset GBS neonatal sepsis (EONS), which represents nearly 80% of all GBS neonatal syndromes, is usually a consequence of vertical transmission during labor [1, 2, 3]. Intrapartum antibiotic prophylaxis (IAP) can prevent this transmission route, and two main approaches are available to select pregnant women that will be submitted to IAP: the risk-based and the culture-based strategies. The Centers for Disease Control and Prevention [4] recommends that all pregnant women in the USA between the 35^th^ and 37^th^ gestational weeks should be screened for vaginal-rectal GBS colonization, and those with positive cultures should be submitted to IAP. In other countries, such as the United Kingdom and the Netherlands, IAP is administered based on the presence of clinical risk factors (such as preterm labor, premature or prolonged rupture of membranes, GBS bacteriuria, previous infant with GBS disease) [5]. The Brazilian Society for Pediatrics [6, 7] recommends the culture-based policy since 2011, but adhesion to these guidelines seems to be very low (around 20%) in Brazil [5].

GBS serotyping is based on antigenic differences of the polysaccharide capsule [8]. Currently, ten different capsular types are recognized, including Ia, Ib, II-IX [9]. The classification in serotypes is widely used for epidemiological purposes and pathogenicity studies and constitutes a valuable tool to predict the impact of putative polysaccharide-based GBS vaccines. Main serotype-based vaccine candidates currently under clinical trials comprise up to five of the ten capsular types described to date [10].

*S*. *agalactiae* is still considered uniformly susceptible to penicillin, although isolates with reduced susceptibility to this drug have been sporadically reported since 2008 [11]. The use of clindamycin or erythromycin was recommended as alternatives in IAP for penicillin-allergic women with high risk of anaphylaxis or when therapeutic failure is suspected [4]. However, increasing rates of clindamycin and erythromycin resistance have been detected in several regions of the world, including Europe [12, 13], Asia [11, 14], North America [15, 16] and South America [17–19]; for this reason, clindamycin does no longer constitute an empiric reliable alternative [5].

In general, data on the occurrence of GBS colonization and distribution of GBS serotypes and antimicrobial susceptibility profiles among pregnant women living in different Brazilian locations are still largely unknown, as the information available is usually related to small groups of patients and short-term observations. In the present study, we evaluated the occurrence, serotype distribution and antimicrobial susceptibility profiling of GBS isolates recovered from pregnant women seeking medical assistance at a public maternity in Rio de Janeiro State, Brazil, during a period of 8 years, and analyzed the association of clinical, social and demographic aspects with GBS colonization.

## Material and methods

### Population included in the study

A total of 3,647 pregnant women between the 35^th^ and 37^th^ gestational weeks, seeking medical attention at a public maternity in Rio de Janeiro State between March 2008 and December 2015, were enrolled in the present study. The maternity is located in a major metropolitan area of Rio de Janeiro State, in the Southeastern region of Brazil. Rio de Janeiro is the third most populated state in the country, and it can be considered as representative of the ethnic, social and economic diversity of the Brazilian population due to the historic high flow of immigration [20, 21]. Clinical and socio-demographic information about the patients was gathered by the hospital medical staff as part of the regular procedures for patient assistance. Clinical aspects investigated included presence of vaginal discharge, preterm birth, urinary tract infections, use of antibiotics during pregnancy, maternal pathology, history of previous neonatal death, history of previous neonatal GBS infection and allergy to penicillin. Socio-demographic data included ethnicity, marital status, scholarship level and place of birth. Written informed consent was obtained from each participant. This study was approved by the research Ethical Committee from University Hospital Clementino Fraga Filho of the Federal University of Rio de Janeiro (UFRJ) under number 219/05.

### Collection of clinical specimens and detection of GBS

A single ano-vaginal specimen was collected from each patient by an attending physician. Either a sterile conventional cotton swab or a flocked swab (Copan Diagnostics Inc., Murrieta, CA, USA) was initially introduced in the middle third of vaginal region and later in the rectum through the anal sphincter, according to CDC recommendations (2010). Each swab was then inoculated in 3 ml of selective Todd-Hewitt broth (sTHB; Plast Labor, Rio de Janeiro, RJ, Brazil) supplemented with nalidixic acid (15 μg/mL; Sigma-Aldrich, St. Louis, MO, USA) and gentamicin (8 μg/mL; Sigma-Aldrich) [9]. After incubation for 18–48 h at 36°C under aerobic atmosphere, an aliquot of each sTHB culture was sub-cultured on 5% sheep blood agar plates (Plast Labor) and incubated for 18–24 h under the same conditions. Colonies with expected morphological and hemolytic patterns on 5% sheep blood agar plates were submitted to identification by conventional procedures, including Gram staining, catalase and CAMP production testing [9]. Serological grouping was performed by using a commercial latex agglutination test (Slidex Strepto Kit, bioMerieux, France), according to the manufacturer’s instructions.

### Determination of capsular types

Serotypes were determined by the Ouchterlony double immunodiffusion method, after HCl extraction of capsular polysaccharides, and using specific antisera (gently provided by the Centers for Disease Control and Prevention, CDC, GA, USA) against types Ia-VIII [8, 9].

### Antimicrobial susceptibility testing

The isolates were tested for susceptibility to ampicillin, chloramphenicol, clindamycin, erythromycin, levofloxacin, tetracycline and vancomycin (Oxoid, Basingstoke, United Kingdom) by the disk diffusion method, according to the CLSI guidelines and interpretative criteria [22]. MLS_B_ phenotypes were determined by the double-disk diffusion test.

### Statistical analysis

Statistical analysis was performed using the two-way ANOVA with assistance of the GraphPad Prism 6 software (GraphPad Software, La Jolla, CA, USA). p-values ≤ 0.05 were considered statistically significant.

## Results

**Fig 1:**
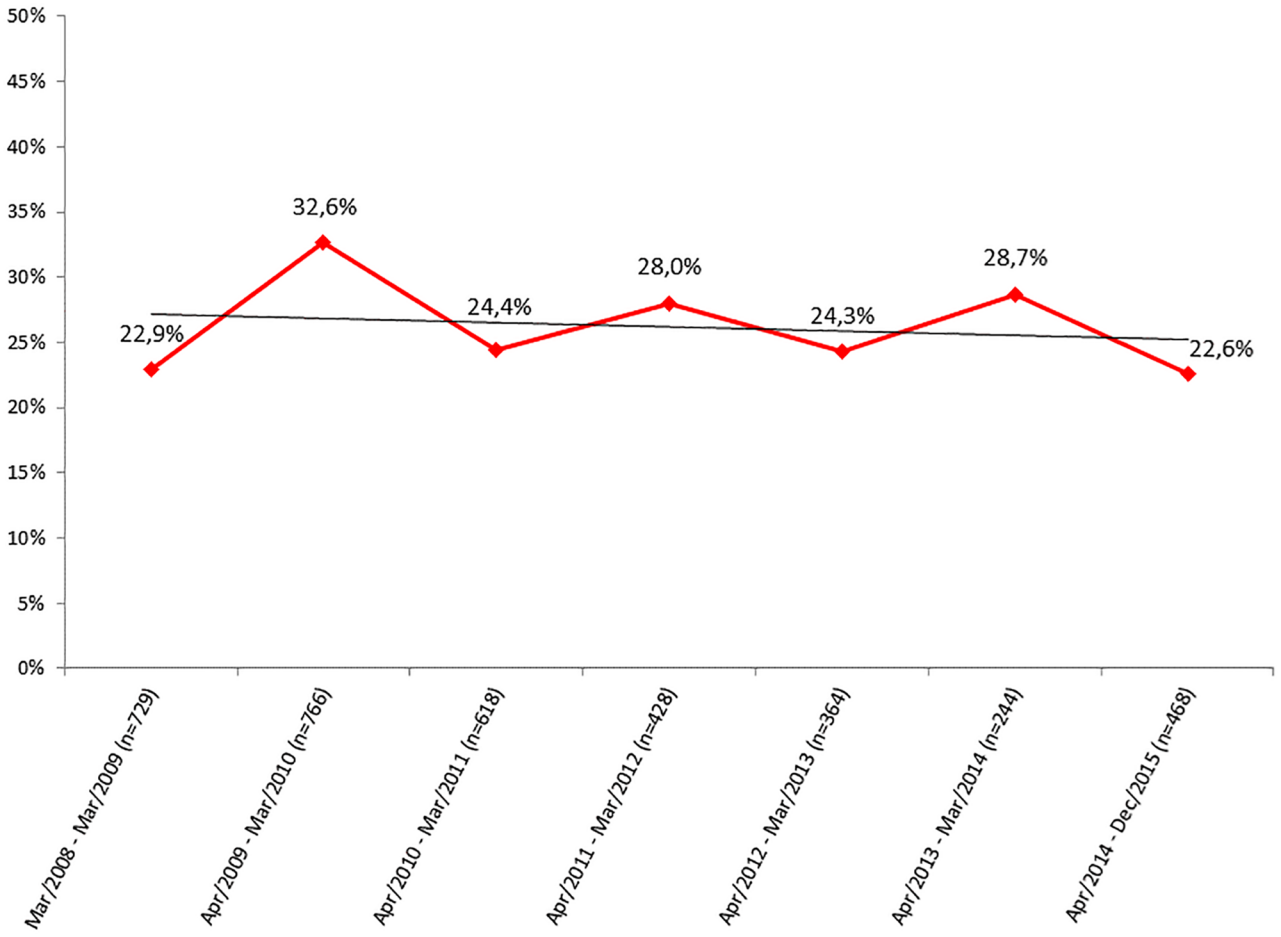
Percentages of GBS carriage among 3,647 pregnant women living in Rio de Janeiro, Brazil, over a period of eight years. The red line indicates relative percentages in each period and the black line represents the tendency line.

GBS colonization was detected in 956 (26.2%) of the 3,647 pregnant women evaluated, and no significant fluctuations in the colonization rates were observed throughout the period of time investigated (Fig 1; p = 0.0693).

The distribution of clinical, social and demographic characteristics among the population investigated, according to the presence or absence of GBS colonization, is shown in Table 1. These data are related to those pregnant women from whom all the specified information was available (a total of 3,369 out of the 3,647 pregnant women enrolled in the present study). The age of the participants ranged from 12 to 48 years old with a mean of 28.26 (standard deviation of ± 6.2). Cephalexin and nitrofurantoin were the antimicrobials most commonly used during pregnancy for treatment of different conditions, followed by penicillin and ceftriaxone. Arterial hypertension and gestational diabetes were the most frequent maternal pathologies observed. Concomitant infections, such as HIV, syphilis, toxoplasmosis and HPV, were also reported. Eight patients reported previous neonatal death due to GBS infections, and three of them were colonized by GBS in the current pregnancy. Among clinical, social and demographic aspects evaluated, presence of vaginal discharge was the only characteristic statistically associated with GBS colonization (p = 0.003), although a trend for an association between white ethnicity and lower GBS colonization rates was observed (p = 0.057). One-hundred twenty-six (13.7%) of the 956 colonized women did not present any clinical risk factor associated with GBS carriage, including previous infant with GBS infection, GBS bacteriuria, premature or prolonged rupture of membranes and premature labor.

**Table 1:** Distribution of clinical, social and demographic aspects according to the presence or absence of *Streptococcus agalactiae* colonization among 3,369 pregnant women enrolled in the present study.

Aspects evaluated	Number of pregnant women GBS-positive total = 753	Number of pregnant women GBS-negative total = 2616	p-value^a^
**Clinical Aspects**			
Presence of vaginal discharge			
Yes	358	1129	
No	395	1487	0.003
Preterm birth			
Yes	29	91	
No	724	2525	0.815
Urinary tract infection diagnosed			
Yes	175	604	
No	578	2012	0.675
Use of antibiotics during pregnancy			
Yes	194	648	
No	559	1968	0.673
Maternal pathology			
Yes	112	419	
No	641	2197	0.638
History of previous neonatal death			
Yes	40	86	
No	713	2530	0.125
History of neonatal GBS infection			
Yes	3	5	
No	750	2611	0.588
**Social and demographic aspects**			
Race or skin color			
White	291	1023	
Non-white	462	1593	0.057^b^
Marital status			
Married	223	673	
Single	490	1847	
Others	40	96	0.299
Level of education			
Elementary School	247	622	
High School	426	1789	
Undergraduation School	80	205	0.908
Place of birth			
North region	5	28	
Northeast region	246	613	
Midwest region	3	11	
South region	2	20	
Southeast region^c^	25	79	
Rio de Janeiro	475	1	
Other countries^d^	2	12	0.478

^a^p-values < 0.05 were considered statistically significant.

^b^p-value refers to the comparison between white and black ethnicities only, since only three pregnant women were of other ethnicities.

^c^Southeast region except Rio de Janeiro State, which is shown separately.

^d^Other countries included Angola, Argentina, Australia, Bolivia, Chile, China, Colombia, Cuba, France, Paraguay, Peru, Senegal and Uruguay. GBS-positive pregnant women comprised one from Chile and one from China.

Among the 956 GBS isolates recovered throughout the period of study, 689 (72.1%) were available for serotyping and antimicrobial susceptibility testing (the remaining 267 were lost or became non-viable), and they represented 49.8 to 100.0% of the isolates recovered in each two-year period of time evaluated (Table 2).

**Table 2:** Number of *Streptococcus agalactiae* isolates analyzed according to the period of time included in the study.

Period of time	Total number of GBS isolates	Number (%) of GBS isolates submitted to serotyping and antimicrobial susceptibility testing
2008–2009	363	240 (66.1%)
2010–2011	277	138 (49.8%)
2012–2013	194	194 (100%)
2014–2015	122	117 (95.9%)
2008–2015	956	689 (72.1%)

**Fig 2:**
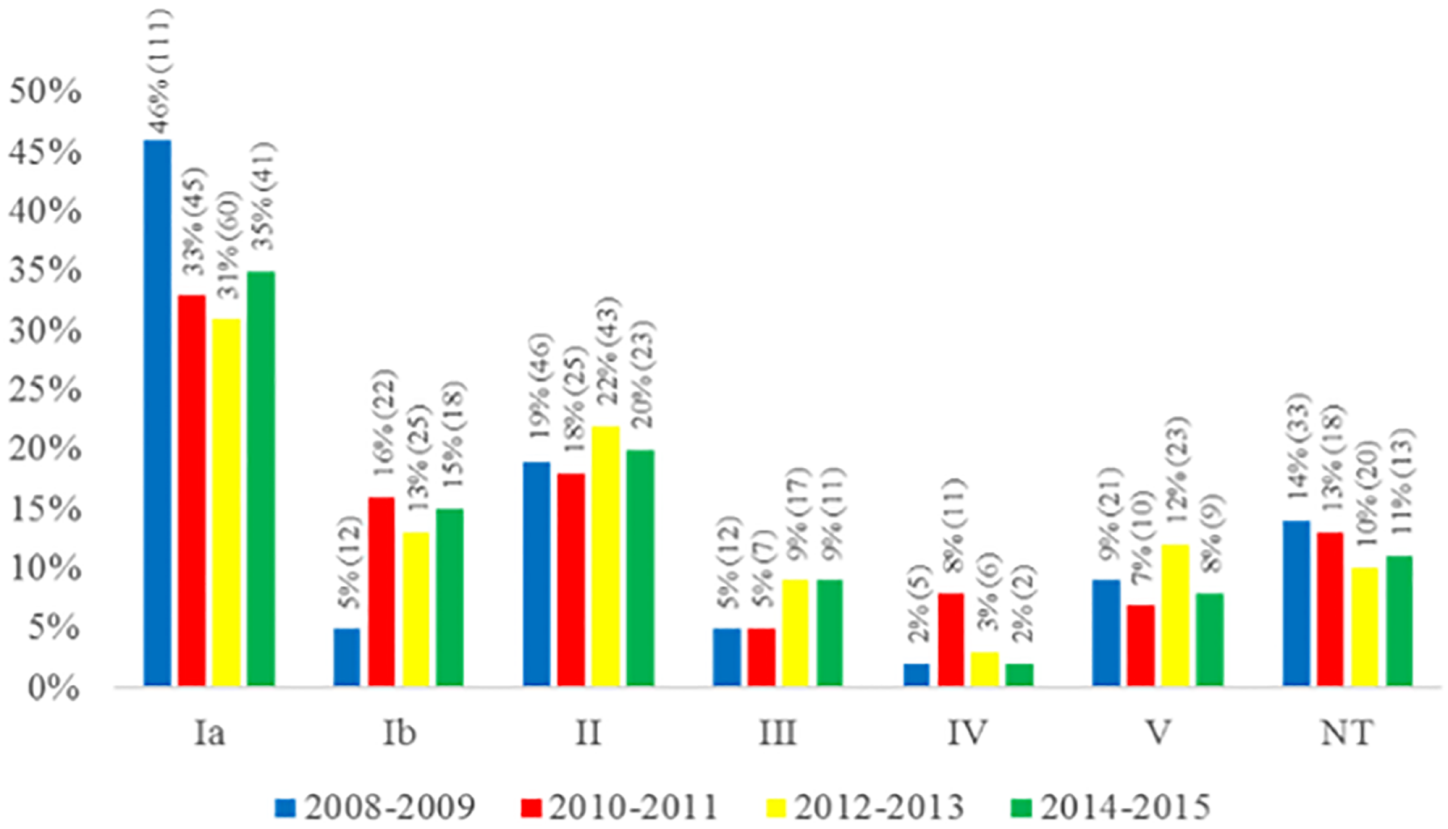
Distribution of serotypes among 689 *Streptococcus agalactiae* isolates recovered from colonized pregnant women living in Rio de Janeiro, Brazil, according to the period of time investigated.

Overall, 257 (37.3%) out of 689 isolates belonged to serotype Ia, 77 (11.2%) to serotype Ib, 137 (19.9%) to serotype II, 47 (6.8%) to serotype III, 24 (3.5%) to serotype IV and 63 (9.2%) to serotype V. Eighty-four (12.1%) isolates were nontypeable (NT). Serotypes VI, VII and VIII were not detected. The distribution of serotypes throughout the period of time included in the study is shown in Fig 2. No significant fluctuations on the frequencies of serotypes were detected throughout the period of investigation, except for serotypes Ia and Ib, which slightly decreased and increased, respectively, from 2010 onwards.

**Fig 3:**
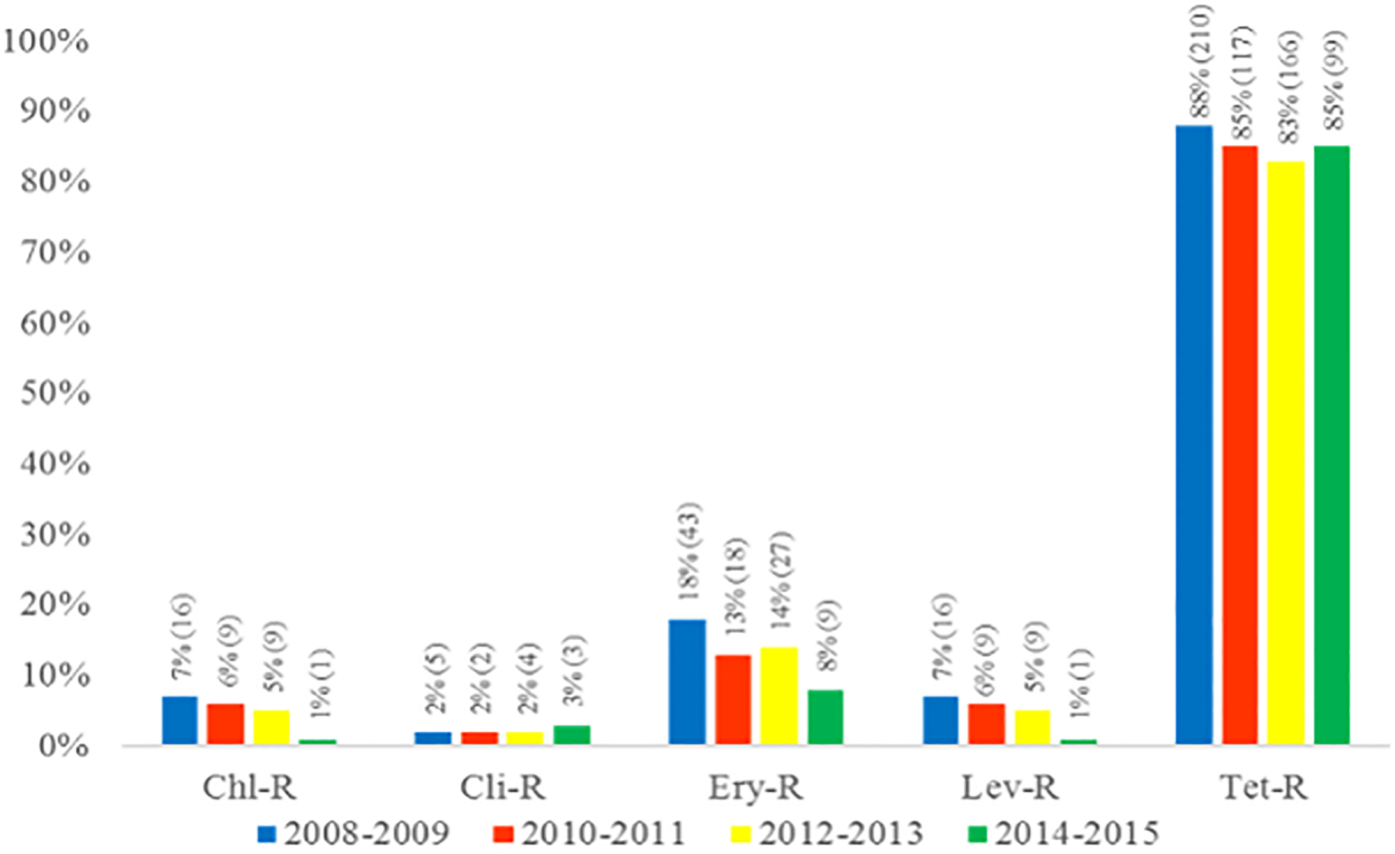
Distribution of antimicrobial resistant profiles among 689 *Streptococcus agalactiae* isolates recovered from colonized pregnant women living in Rio de Janeiro, Brazil, according to the period of time investigated. Chl-R, Chloramphenicol-resistant isolates; Cli-R, Clindamycin-resistant isolates; Ery-R, Erythromycin-resistant isolates; Lev-R, Levofloxacin-resistant isolates; Tet-R, Tetracycline-resistant isolates.

All 689 GBS isolates evaluated were susceptible to ampicillin and vancomycin. Resistance to chloramphenicol and levofloxacin was observed in thirty-five (5%) isolates. Five hundred and ninety-two (86%) isolates showed resistance to tetracycline. Resistance to erythromycin and clindamycin was observed in ninety-seven (14%), and fourteen (2%) isolates, respectively. The distribution of antimicrobial resistance profiles among GBS isolates throughout the period of study did not show any significant fluctuations (Fig 3). Most of the erythromycin-resistant isolates (74/97) presented the M phenotype, while fourteen showed the constitutive MLS_B_ phenotype (cMLSB) and nine had the induced phenotype (iMLSB). No evident association between antimicrobial susceptibility profile and serotype was detected (Table 3). Characteristics of 689 GBS isolates analyzed for serotyping and antimicrobial susceptibility are presented in the Supplementary S1 Table.

**Table 3:** Antimicrobial susceptibility profiles among 689 *Streptococcus agalactiae* isolates recovered from colonized pregnant women in Rio de Janeiro, Brazil.

Antimicrobial susceptibility profile^a^					Number (%) of isolates	Serotype (Number of isolates)	Phenotype^b^
Chl	Cli	Ery	Lev	Tet			
S	S	S	S	R	495 (71.8%)	Ia (220); Ib (54); II (78); III (30); IV (24); V (55), NT (34)	-----
S	S	R	S	R	48 (7%)	Ia (8); Ib (9); II (6) III (11), NT (5)	M (39)
						Ia (7); V (2)	iMLS_B_ (9)
S	S	S	S	S	97 (14.1%)	Ia (15); Ib (8); II (46), NT (28)	----
S	R	R	S	R	14 (2.1%)	II (5); III (6); V (3)	cMLS_B_ (14)
R	S	R	R	R	35 (5%)	Ia (7), Ib (6), II (2), V (3), NT (17)	M (35)

^a^Chl, chloramphenicol; Cli, clindamycin; Ery, erythromycin; Lev, levofloxacin; Tet, tetracycline.

^b^Phenotype of resistance to macrolides, lincosamines and streptogramin B: M, resistance to macrolides; _C_MLSB, constitutive resistance to macrolides, lincosamines and streptogramin B; _i_MLSB, induced resistance to macrolides, lincosamines and streptogramin B.

## Discussion

In the present study, GBS colonization was detected in 26.2% of the pregnant women attending a maternity located in a major urban area in the State of Rio de Janeiro, Brazil, over a period of 8 years. GBS colonization rates may vary according to the geographic area. Different studies indicate that around 20% of pregnant women are colonized by GBS in the USA [15, 23–25]. In Europe, colonization rates range from 19% to 29% in the Eastern region, from 11% to 21% in the Western region, and from 6% to 32% in the Southern region [26]. In Thailand [27] and South Africa [28], 12% and 21% of pregnant women, respectively, are found to carry this microorganism. Studies performed in different Brazilian locations show the occurrence of GBS colonization among pregnant women at rates ranging from 10% to 29% [29–32]. The first report available from Rio de Janeiro, in 1982, showed a GBS colonization rate of 25.2% among pregnant women [33], while a more recent study, conducted with HIV-positive pregnant women in 2011, revealed a rate of 32.2% [34]. Compared to the rate found in the present study, these data indicate that GBS colonization rates in Rio de Janeiro did not fluctuated significantly over the last thirty-five years.

Certain clinical, social and demographic aspects have been previously associated with a higher risk of GBS carriage and development of EONS. In the present study, presence of vaginal discharge was the only characteristic statistically associated with a higher occurrence of GBS colonization, although a strong trend between white pregnant women and lower occurrence of GBS colonization was also seen. Likewise, in a study performed in Santa Catarina, a state located in the South region of Brazil, presence of vaginal discharge and Afro-American ethnicity were characteristics associated with higher prevalence of GBS colonization among pregnant women [35]. In addition, in a study conducted in Ceará, a state located in the Northeast region of Brazil, belonging to white ethnicity was the only characteristic associated with lower prevalence of GBS colonization among 213 pregnant women investigated from 2008 to 2010 [36].

Currently, there is no international consensus as to whether IAP is best achieved through risk-based or culture-based approaches. Reasons why the risk-based strategy is implemented in some places include that culture-based method might not be affordable and/or that risk-based strategy might lead to a lower number of pregnant women exposed to widespread use of antibiotics [5]. If the risk-based approach was considered solely in the present study, a similar percentage of pregnant women would have been submitted to IAP (830/3,369; 24.6%). However, nearly 14% of women known to be colonized by GBS by the culture-based approach would have been excluded from IAP recommendation. These observations suggest that, at least regarding the population analyzed in the present study, the culture-based method seemed to be superior in preventing GBS neonatal diseases since it would not significantly increase the number of pregnant women submitted to antibiotic therapy and would cover a larger number of women who were actually colonized by GBS.

The capsular polysaccharide is a major *S*. *agalactiae* virulence factor, allowing the bacteria to evade the host immune system [25], besides being the target of the major vaccine proposals currently being evaluated [1, 37]. The most common capsular types in this study were Ia and II, together accounting for 57.2% of 689 GBS isolates investigated, while serotypes Ib, III, IV and V were represented in lower percentages ranging from 3.5 to 11.1%. The distribution of serotypes may vary according to several factors, including the geographic region, clinical source of GBS strain, and period of time. Serotypes Ia, III and V are usually the most common in the United States, Europe and Australia [1, 5, 38]. In the present study, the distribution of serotypes was consistent with results of previous reports from Brazil [18, 39], indicating that serotype Ia is the most frequent among GBS isolates recovered from colonization or infection cases in individuals of different ages, occurring at rates of 23 to 38%, followed by serotype II with rates around 15%. Serotype IV was the least frequent in the present study, as it has also been observed in other Brazilian studies [40, 41], with rates ranging from 1 to 5%. Only in Paraná State, in the South of Brazil, this serotype is commonly detected, being described as the third most prevalent [39]. Regarding other serotypes, including Ib, III and V, and non-typeable (NT) isolates, the rates found in the present study are in accordance with previous reports from Brazil [18, 39–41]. Nevertheless, some of the NT isolates in the present study might actually represent encapsulated strains that were not properly detected, not only because serotype IX antiserum was not available for testing, but also because genotyping methods for determining the capsular type were not available.

Moreover, considering the panorama of serotype distribution in the present study, estimated coverage of the main serotype-based GBS vaccines currently under clinical trials would be of 65.2% for the trivalent CRM_197_ conjugate vaccine (targeting serotypes Ia, Ib and III; Novartis) [42] and 84.3% for pentavalent vaccine (targeting Ia, Ib, II, III, and V; Pfizer). Therefore, monitoring the distribution of capsular types among strains circulating in different areas is important not only for elucidating the biology and epidemiology of *S*. *agalactiae* but also for evaluating the potential impact of vaccine strategies according to the peculiarities of each geographic area. This is of particular importance when serotypes not included in vaccine schemes tend to emerge after vaccine introduction; this was the case for pneumococcal conjugate vaccines and for the *Haemophilus influenza* vaccine worldwide [43, 44].

The uniform susceptibility of GBS to beta-lactam antibiotics detected in the present study is in agreement with previous findings from different locations [11, 14, 15, 18, 19, 39–41]. However, reduced susceptibility has been sporadically reported elsewhere [11], underscoring the importance of continuous surveillance of this characteristic among GBS isolates. The rates of resistance to erythromycin (14%) and clindamycin (2%) found in this study are, in general, in accordance with those observed in previous studies conducted in Brazil and in other Latin American countries [17, 18, 39, 41]. Moreover, antimicrobial resistance rates were shown to have no fluctuations over the period of eight years investigated. On the other hand, increasingly higher erythromycin resistance rates have been detected in Asia, Europe, United States and Canada in the last years [11, 13–16]. Our data suggest that, despite of the relatively low resistance rates still detected in Brazil, use of erythromycin and clindamycin as alternative drugs for treating GBS infections in individuals with penicillin allergy should be supported by routine susceptibility testing.

As a limitation of the study, results regarding serotype distribution and antimicrobial susceptibility profiling were obtained from 689 of the 956 GBS isolates recovered from pregnant women. Since characterization of the isolates was not performed in parallel with isolation from clinical samples and preliminary identification, some GBS strains were lost during storage period, especially those isolated in the first years of the study (2008–2011). Nevertheless, the fraction analyzed represented more than 70% of the total number of isolates, and at least nearly 50% of the isolates in each two-year period, being almost fully representative of all isolates during the last four years included in the study (2012–2015).

In conclusion, the present report provides unprecedented volume of data on GBS characteristics among a large population of pregnant women living in Brazil during a long-term period, serving as a basis for assessment of the potential coverage of upcoming vaccines and for improving prevention and treatment strategies that effectively decrease GBS colonization at the moment of labor and, consequently, occurrence of neonatal diseases.

## Supporting information

S1 Table Characteristics of 689 *Streptococcus agalactiae* isolates recovered from pregnant women in Brazil in the present study. (XLSX)
